# New and Emerging Therapies in the Management of Bladder Cancer

**DOI:** 10.12688/f1000research.26841.1

**Published:** 2020-09-16

**Authors:** Chelsea K. Osterman, Matthew I. Milowsky

**Affiliations:** 1 Department of Medicine, Division of Oncology, University of North Carolina School of Medicine, Houpt Physician’s Office Building, 170 Manning Drive, CB #7305, Chapel Hill, NC, 27599, USA; 2 Lineberger Comprehensive Cancer Center, University of North Carolina, Chapel Hill, NC, USA

**Keywords:** urothelial carcinoma, metastatic, muscle-invasive bladder cancer, immunotherapy, targeted therapy, antibody-drug conjugate

## Abstract

The treatment landscape for bladder cancer has undergone a rapid evolution in the past five years with the approval of seven new agents. New classes of medications have improved outcomes for many patients who previously had limited treatment options, but there is still much to learn about how to optimize patient selection for these agents and the role of combination therapies. The aims of this review are to discuss these newly approved agents for bladder cancer and to feature promising drugs and combinations—including immune checkpoint inhibitors, targeted therapies, and antibody–drug conjugates—that are in development.

## Introduction

Bladder cancer is the sixth most common malignancy in the US, where over 80,000 new cases are diagnosed per year ^1^. Non-muscle-invasive bladder cancer (NMIBC) is typically managed with local therapy, including transurethral resection of bladder tumors (TURBT) and intravesical bacillus Calmette–Guérin (BCG) or chemotherapy. NMIBC has an excellent 5-year overall survival (OS) of 70 to 96% ^1^. However, there is also a high rate of recurrence and potential for disease progression ^2^. For muscle-invasive bladder cancer (MIBC), survival outcomes are significantly decreased and treatment, including cystectomy with perioperative chemotherapy or tri-modality therapy (TMT) that includes TURBT, chemotherapy, and radiation therapy, is more aggressive, whereas metastatic disease is generally managed with palliative systemic therapy and has a 5-year OS of about 5% ^1^.

Platinum-based chemotherapy has been the first-line treatment for metastatic bladder cancer for over 20 years. Overall response rates (ORRs) range from 40 to 50% but this response is generally short-lived ^3^. Until recently, options for second-line treatment or platinum-ineligible patients have been limited. However, since 2016, seven new agents have been approved by the US Food and Drug Administration (FDA) for locally advanced (LA) or metastatic urothelial carcinoma (mUC) and this has dramatically changed the treatment landscape. The aims of this review are to highlight these newly approved therapies and to discuss promising new treatment strategies for bladder cancer that are on the horizon.

## Immune checkpoint inhibitors

The introduction of immunotherapy with agents targeting programmed cell death protein 1 or its ligand (anti-PD-[L]1) marked an important turning point in the management of bladder cancer. Currently, five anti-PD-(L)1 drugs are approved by the FDA for urothelial carcinoma: atezolizumab, avelumab, durvalumab, nivolumab, and pembrolizumab. Following initial success in the mUC setting, numerous trials now use these and other anti-PD-(L)1 agents alone and in combination across the continuum of bladder cancer.

### Immunotherapy for metastatic urothelial carcinoma post-platinum

All five of the anti-PD-(L)1 agents for urothelial carcinoma are currently approved by the FDA as treatment for LA/mUC patients who have disease progression during or following platinum-based chemotherapy or within 12 months of neoadjuvant or adjuvant treatment for localized disease with platinum-based chemotherapy. In the trials leading to their approval ^4– 8^, ORRs across all patients ranged from 15% with atezolizumab in IMvigor210 ^6^ to 21.1% with pembrolizumab in KEYNOTE-045 ^8^. Median OS ranged from 6.5 months with avelumab ^4^ to 18.2 months with durvalumab ^5^. Importantly, the phase III KEYNOTE-045 trial of pembrolizumab now has over 2 years of patient follow-up demonstrating a continued OS benefit over second-line chemotherapy with median OS of 10.1 months with pembrolizumab and 7.3 months with chemotherapy (hazard ratio [HR] 0.70, 95% confidence interval [CI] 0.57–0.85) ^9^. The IMvigor211 study was a similarly designed phase III randomized trial comparing atezolizumab versus chemotherapy. However, the primary endpoint, OS, was tested hierarchically in prespecified populations—that is, IC2/3 (PD-L1 expression on at least 5% of tumor-infiltrating immune cells), followed by IC1/2/3, and then the intention-to-treat population. In the IC2/3 population, there was no significant difference in median OS (atezolizumab 11.1 months versus chemotherapy 10.6 months; HR 0.87, 95% CI 0.63–1.21), precluding further formal statistical analyses in the other prespecified populations and thereby resulting in an overall negative study ^10^.

Investigation into the use of immunotherapy combinations post-platinum is ongoing, but early data are promising ( Table 1). The phase II CheckMate 032 trial compared nivolumab monotherapy with nivolumab 3 mg/kg plus ipilimumab 1 mg/kg (NIVO3+IPI1) and nivolumab 1 mg/kg plus ipilimumab 3 mg/kg (NIVO1+IPI3) ^11^. In PD-L1 unselected patients, ORR ranged from 25.6% with nivolumab alone to 38% with NIVO1+IPI3. In patients with PD-L1 expression of at least 1%, ORRs were 26.9% with nivolumab alone but 58.1% with NIVO1+IPI3. Furthermore, median OS was 9.9 months with nivolumab alone and 15.3 months with NIVO1+IPI3 across all patients but was 12.9 and 24.1 months, respectively, in patients with PD-L1 expression of at least 1%. Although grade 3 or 4 treatment-related adverse events were more common with NIVO1+IPI3 compared with nivolumab (39.1% versus 26.9%), these results suggest that combination therapy may provide a significant benefit over monotherapy, particularly for patients whose tumors express PD-L1.

**Table 1.** Selected trials in locally advanced/metastatic urothelial carcinoma with available results.

Trial	Phase	Inclusion criteria	Experimental arm(s)	Patients enrolled	ORR, percentage	DCR, percentage	Median follow-up, months	mPFS, months	mOS, months	mDOR, months	Status
KEYNOTE-045 NCT02256436	III	LA/mUC with progression post- platinum	Pembrolizumab	270	21.1	38.5	27.7	2.1 HR 0.96 (0.79–1.16)	10.1 HR 0.70 (0.57–0.85)	NR	Results published
			Chemotherapy (paclitaxel, docetaxel, or vinflunine)	272	11	44.9	27.7	3.3	7.3	4.4	
CheckMate 032 NCT01928394	I/II	LA/mUC with progression post- platinum or refused chemo	Nivolumab 3 mg/kg (NIVO3)	78	25.6	52.5		2.8	9.9	30.5	Results published
			Nivolumab 3 mg/kg + ipilimumab 1 mg/ kg (NIVO3+IPI1)	104	26.9	50		2.6	7.4	22.3	
			Nivolumab 1 mg/kg + ipilimumab 3 mg/ kg (NIVO1+IPI3)	92	38	63		4.9	15.3	22.9	
IMvigor211 NCT02302807	III	LA/mUC with progression post- platinum (analysis of IC2/3 population)	Atezolizumab	116	23	43		2.4 HR 1.01 (0.75–1.34)	11.1 HR 0.87 (0.63–1.21)	15.9	Results published
			Chemotherapy (paclitaxel, docetaxel, or vinflunine)	118	22	54		4.2	10.6	8.3	
IMvigor130 NCT02807636	III	1L mUC, platinum- eligible	Arm A: atezolizumab + PBC	451	47		11.8	8.2 HR 0.82 (0.7–0.96)	16 HR 1.02 (0.83–1.24)		Preliminary results presented
			Arm B: atezolizumab monotherapy	362	23						
			Arm C: placebo + PBC	400	44		11.8	6.3	13.4		
PIVOT-02 NCT02983045	I/II	1L mUC, cisplatin- ineligible or refuses	NKTR-214 + nivolumab	34	48	70					Preliminary results presented
HCRN GU14-182 NCT02500121	II	LA/mUC with at least SD on 1L PBC	Maintenance pembrolizumab	55	23	58	12.9	5.4 HR 0.65	22 HR 0.91 (0.52–1.59)		Results published
			Placebo	52	10	39	12.9	3.0	18.7		
Javelin Bladder 100 NCT02603432	III	LA/mUC with at least SD on 1L PBC	Maintenance avelumab + BSC	350	9.7	41.1		3.7 HR 0.62 (0.52–0.75)	21.4 HR 0.69 (0.56–0.86)		Preliminary results presented
			BSC alone	350	1.4	27.4		2.0	14.3		
BLC2001 NCT02365597	II	mUC with progression post- chemotherapy and *FGFR2/3* alteration	Erdafitinib	101	40		24	5.5	11.3	6	Results published
NCT01004224	I	LA/mUC with progression post-platinum or contraindication to PBC and *FGFR3* alteration	Infigratinib	67	25.4	64.2		3.75	7.75	5.06	Results published
NCT02122172	II	LA/mUC with progression post- platinum	Afatinib	23	8.6	39		1.4	5.3		Results published
NCT02236195	II	LA/mUC with progression post-platinum and *CREBBP* or *EP300* mutation or deletion	Mocetinostat	17	11	33		57 days	3.5		Results published
EV-101 NCT02091999	I	Part A: mUC with progression post-platinum or cisplatin-ineligible Part B: mUC with renal insufficiency Part C: mUC previously treated with anti-PD-(L)1	Enfortumab vedotin Part A: dose escalation Part B/C: dose expansion	112	43		16.4	5.4	12.3	7.4	Part B completed accrual Part A/C results published
EV-201 NCT03219333	II	Cohort 1: LA/mUC previously treated with anti-PD-(L)1 and PBC Cohort 2: LA/mUC previously treated with anti-PD-(L)1 and cisplatin- ineligible	Enfortumab vedotin	125	44	72	10.2	5.8	11.7	7.6	Cohort 1 results published Cohort 2 recruiting
EV-103 NCT03288545		Cohort A: 1L mUC, cisplatin-ineligible	Cohort A: Enfortumab vedotin + pembrolizumab	45	73.3	93.3	11.5	12.3		NR	Cohort A results presented, additional cohorts recruiting
NCT01631552	I/II	LA/mUC with progression after at least 1 prior therapy	Sacituzumab govitecan	45	31			7.3	18.9	12.6	Results presented
TROPHY-U-01 NCT03547973	II	Cohort 1: LA/mUC with progression after PBC and anti- PD-(L)1 Cohort 2: LA/mUC with progression after anti-PD-(L)1 and platinum- ineligible Cohort 3: LA/mUC with progression after PBC	Cohort 1+2: Sacituzumab govitecan Cohort 3: sacituzumab govitecan + pembrolizumab	35	29		4.1				Cohort 1 preliminary results presented Cohort 2+3 recruiting

1L, first line; BSC, best supportive care; DCR, disease control rate; HR, hazard ratio; IC2/3, PD-L1 expression on at least 5% of tumor-infiltrating immune cells; LA, locally advanced; mDOR, median duration of response; mOS, median overall survival; mPFS, median progression-free survival; mUC, metastatic urothelial carcinoma; NR, not reached; ORR, overall response rate; PBC, platinum-based chemotherapy; SD, stable disease.

There is also a newly established role for anti-PD-(L)1 agents as switch maintenance therapy following completion of first-line platinum-based chemotherapy. The phase III JAVELIN Bladder 100 trial randomly assigned 700 LA/mUC patients whose disease did not progress after first-line platinum-based chemotherapy to receive maintenance avelumab plus best supportive care versus best supportive care alone. At the planned interim analysis, patients receiving maintenance avelumab had a significant improvement in median OS over best supportive care alone (21.4 versus 14.3 months; HR 0.69, 95% CI 0.56–0.86) as well as a progression-free survival (PFS) benefit (3.7 versus 2.0 months; HR 0.62) ^12^. These results led to the recent FDA approval of avelumab switch maintenance therapy following first-line chemotherapy in patients with mUC. Similarly, the phase II HCRN GU14-182 study enrolled LA/mUC patients who achieved at least stable disease following first-line platinum-based chemotherapy and randomly assigned them to receive maintenance pembrolizumab versus placebo ^13^. Patients receiving maintenance pembrolizumab demonstrated an improvement in median PFS compared with placebo (5.4 versus 3.0 months; HR 0.65).

### First-line immunotherapy for metastatic urothelial carcinoma

In addition to approval for patients who progress following platinum-based chemotherapy, atezolizumab and pembrolizumab are approved in the first-line setting for LA/mUC. Both agents were initially approved as first-line treatment for cisplatin-ineligible patients on the basis of the phase II IMvigor210 ^14^ and KEYNOTE-052 ^15^ trials. Subsequently, the randomized phase III IMvigor130 trial of atezolizumab and KEYNOTE-361 trial of pembrolizumab enrolled platinum-eligible patients with LA/mUC and no prior systemic therapy to receive atezolizumab/pembrolizumab with or without platinum-based chemotherapy versus platinum-based chemotherapy alone ^16^. In June 2018, interim analyses of these two trials showed that patients with low PD-L1 expression receiving atezolizumab or pembrolizumab monotherapy had decreased survival compared with patients with low PD-L1 expression who received platinum-based chemotherapy, leading to a change in drug approval ^16^. Currently, atezolizumab and pembrolizumab are indicated as first-line treatment for LA/mUC patients who are cisplatin-ineligible and whose tumors express PD-L1 or patients who are not eligible for any platinum therapy regardless of PD-L1 status. It is important to note that the final analysis of either trial has not been published, but results from IMvigor130 have been presented. Similarly, the phase III DANUBE trial compared durvalumab monotherapy or durvalumab plus tremelimumab versus platinum-based chemotherapy. A press release stated that the study did not meet its primary endpoints for OS in high–PD-L1 patients who received durvalumab or OS in patients who received durvalumab plus tremelimumab regardless of PD-L1 status, but results have not yet been presented or published ^17^.

There is also growing evidence to suggest that the clinical benefit of the combination of immune checkpoint inhibitors with chemotherapy in the first-line setting may be limited. Results from IMvigor130 evaluating the combination of atezolizumab plus chemotherapy demonstrated a PFS benefit over chemotherapy alone (8.2 versus 6.3 months; HR 0.82, 95% CI 0.70–0.96) but this benefit was small and of questionable clinical significance ^18^. One concern regarding the outcome of IMvigor130 is that 40% of patients deemed cisplatin-eligible received carboplatin-based chemotherapy, yet subgroup analysis suggested an overall survival benefit seen only in the cisplatin-treated patients. A recent press release also reported that, in KEYNOTE-361, pembrolizumab plus chemotherapy did not meet the dual primary endpoints for superiority in OS or PFS over chemotherapy alone ^19^. Similar trials are ongoing to further evaluate first-line immunotherapy plus chemotherapy, including CheckMate901 comparing first-line nivolumab plus ipilimumab or standard-of-care chemotherapy versus standard chemotherapy (NCT03036098) and NILE comparing durvalumab plus chemotherapy with or without tremelimumab with chemotherapy alone (NCT03682068) ( Table 2).

**Table 2.** Selected ongoing clinical trials for patients with locally advanced/metastatic urothelial carcinoma.

Trial	Phase	Inclusion criteria	Experimental arm(s)	Comparator arm	Status
**CheckMate901**NCT03036098	III	First-line LA/mUC	Arm A: nivolumab + ipilimumab Arm C: nivolumab + gemcitabine + cisplatin	Arm B: gemcitabine + cis/carboplatin Arm D: gemcitabine + cisplatin	Recruiting
**NILE** NCT03682068	III	First-line LA/mUC	Arm 1: Durvalumab + gemcitabine + cis/ carboplatin Arm 2: Durvalumab + tremelimumab + gemcitabine + cis/ carboplatin	Gemcitabine + cis/carboplatin	Recruiting
**KEYNOTE-361** NCT02853305	III	First-line LA/mUC	Arm 1: Pembrolizumab Arm 2: Pembrolizumab + chemotherapy	Gemcitabine + cis/carboplatin	Completed accrual (press release stating co-primary endpoints not met)
**DANUBE** NCT02516241	III	First-line LA/mUC	Arm 1: Durvalumab Arm 2: Durvalumab + tremelimumab	Gemcitabine + cis/carboplatin	Completed accrual (press release stating primary endpoints not met)
**PIVOT-10** NCT03785925	II	First-line LA/mUC, cisplatin- ineligible	NKTR-214 + nivolumab	None	Recruiting
NCT03473743	Ib/II	Phase Ib: LA/mUC with FGFR alteration and any number of prior lines of therapy Phase 2: LA/mUC with FGFR alteration, no prior systemic therapy, and cisplatin-ineligible	Erdafitinib + cetrelimab	None	Recruiting
NCT02122172	II	LA/mUC treated with prior platinum-based chemotherapy and alteration in *EGFR*, *HER2*, *ERBB3*, or *ERBB4*	Afatinib	None	Recruiting
NCT03854474	I/II	Arm A: LA/mUC with disease progression following platinum-based chemotherapy Arm B: LA/mUC with positive PD-L1 expression and cisplatin- ineligible	Tazemetostat + pembrolizumab	None	Undergoing interim analysis
**EV-103** NCT03288545	II	Cohort D: 1L LA/mUC, cisplatin- eligible Cohort E: 1L LA/mUC, platinum-eligible Cohort G: 1L LA/mUC, platinum-eligible Cohort K: 1L LA/mUC, cisplatin- ineligible	D: EV + cisplatin E: EV + carboplatin G: EV + pembrolizumab + cis/carboplatin K (randomized): EV + pembrolizumab	K (randomized): EV monotherapy	Recruiting
NCT03547973	II	Cohort 1: LA/mUC with progression following platinum and anti-PD-(L)1 Cohort 2: LA/mUC cisplatin- ineligible and post anti-PD-(L)1 Cohort 3: LA/mUC with progression following platinum	1 + 2: sacituzumab govitecan 3: sacituzumab govitecan + pembrolizumab	None	Cohort 1 awaiting final results Cohort 2 and 3 recruiting

1L, first-line; EV, enfortumab vedotin; LA, locally advanced; mUC, metastatic urothelial cancer.

### Immunotherapy for muscle-invasive bladder cancer

Preferred management of MIBC includes neoadjuvant cisplatin-based chemotherapy prior to radical cystectomy (RC), and pathologic complete response (pCR) at cystectomy is associated with increased OS ^20^. Immunotherapy has not yet been approved in the neoadjuvant setting for MIBC, but some preliminary studies show promise ( Table 3). The phase II PURE-01 study enrolled 50 patients with clinical T2-3bN0M0 MIBC and administered three doses of pembrolizumab prior to RC ^21^. At cystectomy, 42% of patients had a pCR and 54% of patients had pathologic downstaging to less than pT2. Among patients with a PD-L1 combined positive score (CPS) of at least 10%, 54.3% achieved a pCR and 65.7% were downstaged to less than pT2 whereas only 13.3% and 26.7% of patients with PD-LI of less than 10% achieved these same outcomes. A significant association between tumor mutation burden (TMB) and pCR was also seen. The similar phase II ABACUS trial administered two cycles of neoadjuvant atezolizumab and observed a pCR rate of 31% ^22^. In contrast to PURE-01, the ABACUS trial found no significant correlation between PD-L1 expression or TMB with pCR or 1-year relapse-free survival rates, but patients with high intraepithelial CD8 ^+^ cells had a significantly higher pCR rate compared with those without CD8 ^+^ cells (40% versus 20%, *P* <0.05). These conflicting biomarker results suggest that additional research is needed to clarify the best biomarker for predicting response to immunotherapy in bladder cancer.

**Table 3.** Selected trials in muscle-invasive bladder cancer with available results.

Trial	Phase	Inclusion criteria	Experimental arm(s)	Patients treated	Patients undergoing RC	pCR, percentage	<pT2, percentage	Median follow-up, months	One-year RFS, percentage	Status
**PURE-01** NCT02736266	II	cT2-3bN0M0 MIBC, plan for RC, any cisplatin eligibility	Neoadjuvant pembrolizumab	50	50	42	54			Results published
**ABACUS** NCT02662309	II	cT2-4aN0M0 MIBC, plan for RC, cisplatin- ineligible or refuses	Neoadjuvant atezolizumab	95	88	31		13.1	79%	Results published
**BLASST-1** NCT03294304	II	cT2-4aN0-1M0 MIBC, plan for RC, cisplatin- eligible	Neoadjuvant nivolumab + gem/cis	41	41	49	66			Results presented
**HCRN GU14-188** NCT02365766	Ib/II	Cohort 1: cT2-4aN0M0 MIBC, plan for RC, cisplatin-eligible Cohort 2: cT2-4aN0M0 MIBC, plan for RC, cisplatin-ineligible	Cohort 1: neoadjuvant pembrolizumab + gem/cis Cohort 2: neoadjuvant pembrolizumab + gemcitabine	40	36	44.4	60	14	80%	Cohort 1: results presented Cohort 2: completed accrual

cTNM, clinical tumor node metastasis stage; gem/cis, gemcitabine + cisplatin; MIBC, muscle-invasive bladder cancer; pCR, pathologic complete response; <pT2, downstaging to non-muscle-invasive disease; RC, radical cystectomy; RFS, relapse-free survival.

Clinical trials have also combined neoadjuvant immune checkpoint inhibitors plus chemotherapy in MIBC. Recent results from the BLASST-1 trial of neoadjuvant nivolumab with gemcitabine and cisplatin demonstrated a pCR rate of 49% and downstaging to less than pT2 in 65.8% of patients ^23^. Similarly, results of a phase Ib/II trial of neoadjuvant pembrolizumab plus gemcitabine and cisplatin reported a pCR rate of 44%, and 61% of patients were downstaged to less than pT2 ^24^. With a median follow-up of 14 months, the estimated 12-month relapse-free survival was 80% and OS was 94%. Together, these studies suggest that immunotherapy will likely have a role in the management of patients with MIBC. Trials are under way to further evaluate perioperative immunotherapy as monotherapy, immunotherapy in rational combinations, and chemo-immunotherapy approaches.

For patients who are either not eligible for or not interested in RC, TMT is an alternative treatment option. TMT involves a maximal transurethral bladder tumor resection followed by concurrent chemoradiation. Several trials are investigating the addition of immunotherapy to chemoradiation in patients with MIBC in an attempt to harness the abscopal effect. KEYNOTE-992 is a phase III trial comparing pembrolizumab with chemoradiation versus placebo with chemoradiation, followed by pembrolizumab or placebo every 6 weeks for up to a year, SWOG/NRG 1806 is evaluating the use of chemoradiation with or without atezolizumab, and another trial is evaluating pembrolizumab and gemcitabine with concurrent radiation therapy (NCT02621151). Results from these trials are not yet available but are eagerly anticipated ( Table 4).

**Table 4.** Selected ongoing clinical trials for patients with muscle-invasive bladder cancer.

Trial	Phase	Inclusion criteria	Experimental arm(s)	Comparator arm	Status
KEYNOTE-905 NCT03924895	III	Cisplatin-ineligible MIBC, fit for RC	Perioperative pembrolizumab + RC + PLND	RC + PLND	Recruiting
AMBASSADOR NCT03244384	III	Muscle invasive or locally advanced urothelial carcinoma post-surgery, ineligible for or declines cisplatin	Adjuvant pembrolizumab	Observation	Recruiting
CheckMate 274 NCT02632409	III	Invasive urothelial cancer post-surgery at high risk of recurrence	Adjuvant nivolumab	Placebo	Completed accrual
IMvigor010 NCT02450331	III	Invasive urothelial cancer post-surgery at high risk of recurrence	Adjuvant atezolizumab	Observation	Awaiting final results (press release stating DFS endpoint not met ^26^)
NCT02845323	II	Cisplatin-ineligible/refusing MIBC, fit for RC	Neoadjuvant nivolumab + urelumab	Neoadjuvant nivolumab	Recruiting
EV-103 NCT03288545	I/II	Cisplatin-ineligible MIBC, fit for RC	Cohort H: Neoadjuvant EV Cohort J: Neoadjuvant EV + pembrolizumab		Recruiting
ENERGIZE	III	Cisplatin-eligible MIBC, fit for RC	Arm B: Neoadjuvant nivolumab + chemo + placebo followed by adjuvant nivolumab + placebo Arm C: Neoadjuvant nivolumab + chemo + BMS-986205 (IDO inhibitor) followed by adjuvant nivolumab + BMS-986205	Arm A: Gemcitabine + cisplatin followed by RC	Recruiting
NCT02690558	II	Cisplatin-eligible MIBC, fit for RC	Neoadjuvant pembrolizumab + gemcitabine + cisplatin		Completed accrual
NEMIO NCT03549715	I/II	Cisplatin-eligible MIBC, fit for RC	Arm A: Neoadjuvant durvalumab + ddMVAC Arm B: Neoadjuvant durvalumab + tremelimumab + ddMVAC		Recruiting
NIAGARA NCT03732677	III	Cisplatin-eligible MIBC, fit for RC	Neoadjuvant durvalumab + gemcitabine + cisplatin followed by adjuvant durvalumab	Neoadjuvant gemcitabine + cisplatin	Recruiting
NCT04228042	I/II	Low-grade UTUC or high- grade UTUC and cisplatin- ineligible	Neoadjuvant infigratinib		Recruiting
NCT02621151	II	Localized MIBC, not a candidate for or declines RC	Tri-modality therapy with maximal TURBT and gemcitabine + pembrolizumab concurrent with EBRT		Recruiting
SWOG 1806 NCT03775265	III	Localized MIBC	Concurrent chemotherapy + atezolizumab + radiation	Concurrent chemotherapy + radiation	Recruiting
KEYNOTE-992 NCT04241185	III	Localized MIBC, opting for bladder preservation	Pembrolizumab + CRT	Placebo + CRT	Recruiting
PROOF 302 NCT04197986	III	Invasive urothelial carcinoma with *FGFR3* alteration at high risk for recurrence following RC or nephrectomy	Adjuvant infigratinib	Placebo	Recruiting

CRT, chemoradiation; ddMVAC, dose-dense methotrexate, vinblastine, doxorubicin, cisplatin; DFS, disease-free survival; EBRT, external beam radiation therapy; EV, enfortumab vedotin; MIBC, muscle-invasive bladder cancer; PLND, pelvic lymph node dissection; RC, radical cystectomy; TURBT, transurethral resection of bladder tumor; UTUC, upper tract urothelial carcinoma.

### Immunotherapy for non-muscle-invasive bladder cancer

The first FDA approval for immunotherapy in the non-metastatic setting came in January 2020 when pembrolizumab was approved for patients with BCG-unresponsive, high-risk NMIBC with carcinoma *in situ* (CIS) with or without papillary tumors who are ineligible for or have chosen not to undergo cystectomy. This approval was based on results from cohort A of the KEYNOTE-057 trial showing a 3-month complete response (CR) rate of 40% with 46% of responses lasting at least 12 months ^25^. Similar results were seen in the phase II SWOG S1605 trial of atezolizumab in BCG-unresponsive NMIBC. Preliminary results from 73 patients showed a 3-month CR rate of 41% and 6-month CR rate of 26% ^27^. Additional trials evaluating the use of immunotherapy in NMIBC are ongoing; these include the phase III KEYNOTE-676 trial of BCG with or without pembrolizumab (NCT03711032), a phase II trial of gemcitabine plus pembrolizumab (NCT04164082), and the phase II ADAPT-Bladder trial comparing durvalumab monotherapy, durvalumab plus BCG, and durvalumab plus external beam radiation (NCT03317158).

## Targeted therapy

One of the most important recent advances in urothelial cancer is the genomic profiling of tumors, which has revealed a number of common genomic alterations ^28– 30^. In an analysis of 412 MIBCs as part of The Cancer Genome Atlas Project, 58 significantly mutated genes were identified ^28^. Clinically relevant alterations in MIBC include changes in the genes for cyclin-dependent kinase inhibitor 2a ( *CDKN2A*), fibroblast growth factor receptor 3 ( *FGFR3*), erythroblastic oncogene B/human epidermal growth factor receptor 2 ( *ERBB2/HER2*), and phosphatidylinositol 3-kinase catalytic subunit alpha ( *PIK3CA*). Additionally, mutations in chromatin-modifying genes are found in up to 83% of patients with urothelial cancer, which has spurred investigation into agents targeting these alterations ^31, 32^.

### FGFR

Erdafitinib is a tyrosine kinase inhibitor of FGFR1–4 and the first targeted therapy approved for mUC. The phase II trial of erdafitinib included 99 patients whose tumor harbored an *FGFR3* mutation or *FGFR2/3* fusion and who had disease progression following chemotherapy ^33^. The confirmed ORR was 40% and an additional 39% of patients had stable disease. A total of 22 patients had previously received immunotherapy with only one achieving a response, yet the response rate for erdafitinib for this subgroup was 59%. At a median follow-up of 24 months, the median PFS was 5.5 months (95% CI 4.0–6.0) and the median OS was 11.3 months (95% CI 9.7–15.2) ^34^. Based on these results, erdafitinib was approved by the FDA in April 2019 for patients with mUC with a susceptible *FGFR2/3* alteration following platinum-containing chemotherapy. Multiple ongoing trials—including a phase II study of erdafitinib alone or in combination with cetrelimab, an anti-PD-1 antibody, and as first-line therapy for cisplatin-ineligible patients with mUC and an FGFR alteration (NCT03473743)—are assessing erdafitinib in other clinical scenarios.

In addition to erdafitinib, other FGFR inhibitors are under investigation. An expansion cohort to the phase I trial of infigratinib included 67 mUC patients who progressed on or had contraindications to platinum-based chemotherapy and whose tumor harbored an alteration in *FGFR3* ^35^. The confirmed ORR was 25.4%, median duration of response was 5.06 months, median PFS was 3.75 months, and median OS was 7.75 months. A subsequent analysis of this same cohort observed that patients with upper tract urothelial cancer (UTUC) had a confirmed ORR of 50% and a disease control rate (DCR) of 100% but that those with urothelial cancer of the bladder (UCB) had an ORR of 22% and a DCR of 59.3% ^36^. Additionally, the median PFS and median OS were 8.54 and 21.82 months for those with UTUC and 3.65 and 7.0 months for those with UCB. Prior work has shown that *FGFR3* alterations are more common in UTUC than UCB (40% versus 26%) ^37^ and thus UTUC may be more amenable to FGFR inhibition. Although this study included a small number of patients with UTUC, these initial results certainly warrant further evaluation and a phase I/II trial of neoadjuvant infigratinib for patients with UTUC is planned (NCT04228042). Additionally, a phase III trial comparing adjuvant infigratinib versus placebo in patients with FGFR3 alterations and high risk for disease recurrence is under way (NCT04197986).

### Chromatin-modifying genes

Chromatin structure can be modified via many mechanisms, including histone acetylation/deacetylation and histone methylation/demethylation, resulting in regulation of gene transcription. Disruption of this process is implicated in the pathogenesis of urothelial cancer and therefore may be a viable target for new therapies, such as histone deacetylase (HDAC) inhibitors and enhancer of zeste homolog 2 (EZH2) inhibitors ^38^. Mocetinostat, a class I/IV HDAC inhibitor, was administered to 17 patients with mUC with progression after platinum-based chemotherapy and an inactivating mutation or deletion in *CREBBP, EP300* ^39^, or both. The ORR was 11% in stage 1 and so the study was terminated. Although mocetinostat did not appear to be effective in this cohort of patients with mUC, it is possible that a different biomarker is needed to predict patient response.

Pre-clinical studies have demonstrated that EZH2 inhibition induces cell death in models of urothelial cancer ^40, 41^. Additionally, the response appears to be enhanced when the EZH2 inhibitor tazemetostat is combined with an anti-PD-1 antibody ^42^. Based on these findings, a phase I/II trial evaluating the combination of tazemetostat plus pembrolizumab in patients with either cisplatin-refractory or cisplatin-ineligible mUC is under way (NCT03854474). Similarly, EZH2 inhibition has been shown to improve the response to anti-CTLA-4 therapy in a murine model of bladder cancer ^43^. This led to the phase Ib/II ORIOn-E trial of the EZH2 inhibitor CPI-1205 combined with ipilimumab, which includes a cohort of patients with mUC (NCT03525795). This trial is currently closed to accrual, but results have not yet been released. Other agents targeting chromatin modification genes appear promising in pre-clinical studies and are anticipated to move into early-phase clinical trials in the near future.

### HER2

Multiple agents targeting HER2, including trastuzumab, lapatinib, and afatinib, have also been tested in patients with urothelial cancer ^44^. Results thus far have been somewhat mixed, possibly partially owing to inclusion of HER2 unselected patients and the discordance in HER2 classification between immunohistochemistry (IHC), fluorescence *in situ* hybridization, and molecular characterization. A phase II study of afatinib in HER2 unselected patients with platinum-refractory mUC found an ORR of 8.6% and a median PFS of 1.4 months for the entire cohort ^45^. However, they also found that 83% (5/6) of patients with *HER2* copy number amplification or *ERBB3* somatic mutations (or both) achieved a PFS of at least 3 months but that 0% of patients without alterations did. Interestingly, no correlation between IHC for ERBB3, HER2, or EGFR and clinical response to afatinib was seen. Additional trials of HER2 targeted agents, including a follow-up study of afatinib in patients with alterations in EGFR, HER2, ERBB3, or ERBB4 (NCT02122172), are ongoing. It remains to be seen whether this will prove to be a viable treatment option in appropriately selected patients.

## Antibody–drug conjugates

Antibody–drug conjugates (ADCs) are a class of cancer therapeutic that link a monoclonal antibody specific for a tumor cell-surface protein with a cytotoxic agent. A number of ADCs have received FDA approval across a wide variety of tumor types, including ado-trastuzumab emtansine for Her2 ^+^ breast cancer, brentuximab vedotin for CD30 ^+^ Hodgkin’s lymphoma, and most recently enfortumab vedotin (EV) for urothelial cancer ^46^.

### Enfortumab vedotin

EV is an ADC targeting Nectin-4, a cell adhesion molecule highly expressed in nearly all urothelial tumors, conjugated to monomethyl auristatin E, a microtubule-disrupting agent ^47^. In the dose expansion portion of the EV-101 trial, 112 mUC patients who failed at least one prior therapy received EV ^48^. The confirmed ORR was 43%, including 5% CR, and the median duration of response was 7.4 months. Subsequently, EV-201 enrolled patients with mUC treated with prior platinum and anti-PD-(L)1 (cohort 1) or treated with prior anti-PD-(L)1 and cisplatin-ineligible (cohort 2) ^49^. Cohort 1 enrolled 125 patients with a confirmed ORR of 44%, including a 12% CR rate. Responses were seen across subgroups including an ORR of 41% in non-responders to prior anti-PD-(L)1 and 38% in patients with liver metastases. Results for cohort 2 have not yet been released.

EV-103 is an ongoing multi-cohort trial of EV alone or in combination with other therapies and includes cohorts of patients with mUC and localized MIBC. Cohort A evaluated EV plus pembrolizumab as first-line treatment for 45 cisplatin-ineligible patients with LA/mUC ^50^. The confirmed ORR was 73.3%, including 15.6% CRs, and the DCR was 93.3%. With a median follow-up of 10.4 months, the median duration of response was not yet reached and the median PFS was 12.3 months. These results are extremely encouraging, particularly for cisplatin-ineligible patients who have limited effective treatment options. Additional study cohorts, including EV plus chemotherapy as first-line treatment for mUC and EV alone or with pembrolizumab as neoadjuvant therapy for localized MIBC (NCT03288545), are ongoing.

### Sacituzumab govitecan

Sacituzumab govitecan (SG) is an ADC employing an anti-Trop-2 antibody conjugated to SN-38, the active metabolite of irinotecan ^51^. Trop-2 is a transmembrane glycoprotein that is important for cell growth and tumorigenesis and that is overexpressed in urothelial cancer. The phase I/II basket trial of SG included 45 mUC patients who had received at least one prior line of systemic therapy ^52^. The ORR was 31%, median duration of response was 12.6 months, median PFS was 7.3 months, and median OS was 18.9 months. The subsequent phase II TROPHY-U-01 trial of SG recently completed accrual for cohort 1, which enrolled 100 mUC patients who progressed after platinum-based therapy and a checkpoint inhibitor ^53^. Preliminary results from 35 patients demonstrated an ORR of 29% and in light of these data the FDA granted SG fast-track designation for mUC in April 2020 ^54^. Accrual to two additional patient cohorts—a cohort of platinum-ineligible patients who progressed following checkpoint inhibitor therapy and a cohort of immune checkpoint inhibitor-naïve patients who will receive SG plus pembrolizumab—is ongoing.

## Summary

The management of bladder cancer has changed dramatically in the past 5 years and is poised to evolve further in the coming years. The approval of seven new drugs during this time has created new options for many patients and in some cases has led to long-term responses. Despite these encouraging successes, however, much work remains to be done.

Considerable excitement has surrounded immune checkpoint inhibitors for bladder cancer, but ORR is still only about 15 to 25% with monotherapy for metastatic disease. Multi-agent therapy, employing different combinations of immunotherapy, chemotherapy, or targeted therapy, may prove to be more efficacious, and further clinical trials testing this strategy are under way. The role of immune checkpoint inhibitors at different disease stages is also undergoing refinement, starting with FDA approval of pembrolizumab in high-risk NMIBC. It is likely only a matter of time until immune checkpoint inhibitors are approved as part of perioperative treatment for patients with MIBC, and evolving data suggest a role for maintenance immunotherapy following induction chemotherapy in metastatic disease.

The discovery of better biomarkers to help select patients who are more likely to respond to certain therapies will also prove important in the years to come. As was seen in the PURE-01 and ABACUS trials, markers such as PD-L1 expression and TMB appear predictive in some cases but not in others, and the possible predictive role of CD8 ^+^ cell expression or gene signature expression requires further elucidation. Many mutations are commonly found in bladder cancer, suggesting that targeted therapy has great potential to influence the treatment landscape. We must continue to work to understand which alterations confer susceptibility to targeted inhibition and what is the best method to detect these alterations. As our knowledge of the biological drivers of carcinogenesis and factors influencing treatment response improves, so too will the outcomes of our patients.
